# Supplementary material for: Performance of the low-cost phenotypic thin-layer agar MDR/XDR-TB Colour Test (first generation, 1G, Color Plate Test) for identifying drug-resistant Mycobacterium tuberculosis isolates in a resource-limited setting
Source: BMC Microbiol. 2025 Oct 10;25:652. doi: 10.1186/s12866-025-04347-z (PMC12512722; doi:10.1186/s12866-025-04347-z)
Supplement: Supplementary file 1 — Supplementary Material 1. [file 12866_2025_4347_MOESM1_ESM.docx]

**Table 1.** Characteristics of the study population.

| **Variable** | **Number** | **percentage** | **95%CI** |  | |
| --- | --- | --- | --- | --- | --- |
| Female sex(versus male) | 43/77 | 56 | 45-67 |  |  |
|  |  |  |  |  |  |
| Age(years) | 77 | 99 |  | Median | 27 |
|  |  |  |  | IQR | 23-34 |
|  |  |  |  | Range | 0.5-70 |
| Hiv seropositive | 5/49 | 10 | 1.7-19 |  | |
|  |  |  |  |  |  |
| Retreatment TB  (versus new cases | 13/75 | 17 | 7.7-26 |  |  |
|  |  |  |  |  |  |
| Fine needle aspirate sample  (versus sputum) | 26/78 | 33 | 23-44 |  |  |
|  |  |  |  |  |  |

Note: as indicated by the “Number” column, some data were missing for some of the 78 samples; CI indicates confidence interval; IQR indicates inter-quartile range; and HIV indicates human immunodeficiency virus.

Isolates, (78) were derived from patients who were 55% male, with a mean age of 30 years (SD=14), and 52 (67%) arose from sputum samples of pulmonary TB patients whereas 26 (33%) were isolated from lymph node aspirates from patients with extra-pulmonary TB. The majority of the samples analysed, 62/78 (79%), were collected from newly diagnosed TB patients; of the remaining 6/78 (7.7%) had treatment failure, 5.1% (4/78) had a prior history of TB treatment with anti-TB medications, 3/78 (3.8%) were relapse and 3/78 (3.8%) had unknown TB treatment outcomes results. 29/78(37%) were unknown where as 10% (5/49) of the *Mtb* isolates were collected from people living with HIV (PLWH), other.
